# Characterization of Argonaute nucleases from mesophilic bacteria *Paenibacillus borealis* and *Brevibacillus laterosporus*

**DOI:** 10.1186/s40643-021-00478-z

**Published:** 2021-12-19

**Authors:** Huarong Dong, Fei Huang, Xiang Guo, Xiaoyi Xu, Qian Liu, Xiao Li, Yan Feng

**Affiliations:** State Key Laboratory of Microbial Metabolism, School of Life Sciences and Biotechnology, Shanghai Jiao Tong University, Shanghai, 200240 People’s Republic of China

**Keywords:** Mesophilic Argonaute protein, Endonuclease, DNA cleavage, Allosteric transcription factors, Detection of small molecule

## Abstract

Thermophilic Argonaute proteins (Agos) have been shown to utilize small DNA guides for cleaving complementary DNA in vitro, which shows great potential for nucleic acid detection. In this study, we explored mesophilic Agos for the detection of small molecule by cooperating with allosteric transcription factors (aTFs). Two Agos from mesophilic bacteria, *Paenibacillus borealis* (*Pb*Ago) and *Brevibacillus laterosporus* (*Bl*Ago), showed nuclease activity for single-stranded DNA at moderate temperatures (37 °C) by using 5′-phosphorylated and 5′-hydroxylated DNA guides. Both Agos perform programmable cleavage of double-stranded DNA, especially in AT-rich regions of plasmid. Furthermore, we developed a simple and low-cost p-hydroxybenzoic acid detection method based on DNA-guided DNA cleavage of Agos and the allosteric effect of HosA, which expands the potential application of small molecule detection by Agos.

**Supplementary Information:**

The online version contains supplementary material available at 10.1186/s40643-021-00478-z.

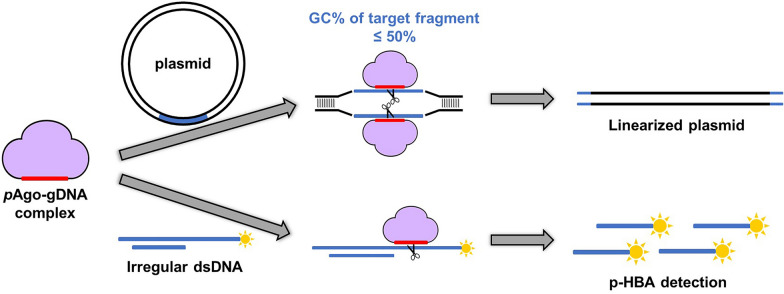

## Introduction

Argonaute proteins (Agos) belong to the PIWI protein superfamily, defined by the existence of the P-element-induced wimpy testis (PIWI) domain, which binds small DNA or RNA guides to specifically recognize or cleave complementary nucleic acid targets (Kirsch et al. 2013; Swarts et al. 2014a, 2014b). Eukaryotic Argonaute proteins (eAgos) are key participants in the RNA interference pathways (Meister 2013; Peters and Meister 2007; Pratt and MacRae 2009) and act as RNA-guided RNA endonucleases (Ketting 2011). Agos from prokaryotes (pAgos) bind single-stranded DNA (ssDNA) guides to specifically cleave complementary DNA, which can mediate host defense against invading nucleic acids in vivo (Enghiad and Zhao 2017; Hegge et al. 2019, 2018; Koonin 2017; Lisitskaya et al. 2018; Makarova et al. 2009; Ryazansky et al. 2018; Swarts et al. 2014a, 2014b). Our group and others have established new methods for nucleic acid detection by taking advantage of the high activity and stability of thermophilic Agos, including *Pf*Ago (*Pyrococcus furiosus*) (Enghiad and Zhao 2017; Liu et al. 2021a; Swarts et al. 2015), *Tt*Ago (*Thermus thermophilus*) (Jolly et al. 2020; Sheng et al. 2014; Swarts et al. 2017), and *Mj*Ago (*Methanocaldococcus jannaschii*) (Willkomm et al. 2017).

Recently, some pAgos from mesophilic bacteria have been reported successively, including *Cb*Ago (*Clostridium butyricum*) (Hegge et al. 2019; Kuzmenko et al. 2019, 2020), *Lr*Ago (*Limnothrix rosea*) (Kuzmenko et al. 2019), *Cp*Ago (*Clostridium perfringen*) (Cao et al. 2019), *Ib*Ago (*Intestinibacter bartlettii*) (Cao et al. 2019), *Se*Ago (*Synechococcus elongatus*) (Olina et al. 2020), and *Km*Ago (*Kurthia massiliensis*) (Liu et al. 2021b). Most of them exert DNA-guided DNA cleavage activity at moderate temperatures and can cleave plasmids with low GC content. Among them, *Cb*Ago, *Cp*Ago, and *Ib*Ago displayed the highest activity of cleaving ssDNA guided by DNA at 37 °C; *Lr*Ago and *Km*Ago displayed the highest activity at 50–55 °C. The reported mesophilic pAgos can only cleave negatively supercoiled plasmid DNA, but not the linearized plasmid. The GC content of the plasmid target fragment also affect the efficiency of mesophilic pAgos-cleaving plasmid. For instance, *Cp*Ago cleaves plasmid fragments with a 59% GC content or lower (Cao et al. 2019). *Km*Ago can cut plasmid fragments with GC content of no more than 53% (Liu et al. 2021b). *Cb*Ago can generate double-stranded DNA (dsDNA) breaks in plasmid fragments with a GC content of 50% or less (Kuzmenko et al. 2019). *Lr*Ago can cut plasmid fragments with a GC content of less than 35% (Kuzmenko et al. 2019). *Ib*Ago can only produce dsDNA breaks in plasmid fragments with GC content of 31% or lower (Cao et al. 2019). These results indicate that the reported mesophilic pAgos rely on the unwinding of dsDNA for targeting and cleavage, especially in AT-rich DNA regions. Since mesophilic Agos possess the potential for biotechnological applications, such as genome editing and detection of DNA-coupled biomarker molecule, it is an attractive target to find mesophilic Agos with high nuclease activities.

p-Hydroxybenzoic acid (p-HBA) is an antiseptic used in foods, medicine, and cosmetics because of its ability to inhibit bacteria and fungi (Soni et al. 2005). Thus, the detection of small molecule, such as antiseptic p-HBA, is of great significance for scientific research, environmental monitoring, food safety, and disease diagnosis (Roy and Ranjan 2016). At this stage, routine detection of p-HBA mainly relies on chromatography, but expensive equipment and cumbersome operations limit the application and promotion of this method. Therefore, several simple and convenient detection methods for small molecule have been developed based on allosteric transcription factors (aTFs). aTFs are regulatory proteins widely distributed in bacteria, usually comprising an effector binding domain (EBD) and a DNA binding domain (DBD). The small molecule effector changes the conformation of aTF by binding to EBD, which can either attenuate or enhance the binding ability of aTF and DNA (Kirsch et al. 2013). aTFs can transfer small molecular signals into DNA signals, which are easy to be detected, making them a valuable biorecognition element for small molecule detection (Li et al. 2016; Libis et al. 2016). Recently, a robust and easy-to-implement signal transduction system, aTF-NAST (aTF-based nicked DNA template-assisted signal transduction), has been developed for the detection of small molecule, utilizing the competition between T4 DNA ligase and aTFs in binding to nicked DNA (Yao et al. 2018). However, this method is relatively time consuming and costly and still requires improvement. A simple and high-throughput platform for the detection of small molecule (uric acid and *p*-hydroxybenzoic acid), designated CaT-SMelor (CRISPR-Cas12a- and aTF-mediated small molecule detector), was developed by combining the trans-cleavage activity of CRISPR-Cas12a (Li et al. 2018) and the allosteric effect of aTFs (Liang et al. 2019), but the cost of RNA reporters is relatively high. To apply this method to daily small molecule detection, it is necessary to develop a simpler and low-cost detection method.

Through phylogenetic tree analysis of *Cb*Ago, we found two pAgos from mesophilic bacteria, *Paenibacillus borealis* (*Pb*Ago) and *Brevibacillus laterosporus* (*Bl*Ago), and studied their biochemical properties of cleaving ssDNA and dsDNA under a wide range of conditions. We also attempted to detect p-HBA based on DNA-guided DNA cleavage of *Pb*Ago/*Bl*Ago and the allosteric effect of HosA (Cao et al. 2018; Liang et al. 2019; Yao et al. 2018). Our study provides new enzyme resources for gene manipulation and shows that the method we developed has great potential for routine small molecule detection for different purposes.

## Materials and methods

### Bacterial strain, plasmid, and medium

The host strain *Escherichia coli* BL21 (DE3) was purchased from Novagen (Madison, WI, USA). The recombinant plasmid pET-28a (+)-*Pb*Ago/*Bl*Ago containing the synthesized codon-optimized Ago gene was constructed (GenScript, China). Luria–Bertani (LB) medium (tryptone 10 g/L, yeast extract 5 g/L, and NaCl 10 g/L) was used for Ago expression.

### Phylogenetic tree and sequence alignment

A similarity search for the *Cb*Ago amino acid sequence was performed in the NCBI database using BLAST, and Ago sequences with high sequence identity were selected and analyzed using MEGA software (version 7.0) (Kumar et al. 2016) to construct a phylogenetic tree. Sequence alignments of the Ago family were carried out using ClustalW (Thompson 1994). For clarity, only the residues forming active sites are displayed.

### Cloning and expression of *Pb*Ago and *Bl*Ago in *E. coli* BL21 (DE3)

The expression vector pET-28a (+)-*Pb*Ago/*Bl*Ago was transformed into the *E. coli* BL21 (DE3) strain to express the recombinant Ago. The positive clones were then propagated overnight in a shaker incubator at 37 °C and 220 rpm in 5 mL of LB medium containing 50 μg/mL kanamycin. After the overnight incubation, the seed culture (1%) was inoculated into a 1 L LB medium containing 50 μg/mL kanamycin at 37 °C and incubated at 220 rpm until an OD_600_ value of 0.6–0.8 was reached. *Pb*Ago and *Bl*Ago expression was induced by the addition of isopropyl-β-D-1-thiogalactopyranoside (IPTG) to a final concentration of 0.5 mM. During the expression, the cells were incubated at 18 °C for 16–18 h with continuous shaking. The cells were harvested through centrifugation for 30 min at 6000 rpm, and the cell pellets were collected for further purification.

### Purification of *Pb*Ago and *Bl*Ago and co-purification of nucleic acids

The harvested cell pellets were resuspended in lysis buffer (20 mM Tris–HCl, 500 mM NaCl, 10 mM imidazole, 2% [v/v] glycerol, 0.05% [v/v] Triton X-100, and pH 8.0) and then disrupted using a high-pressure homogenizer (Gefran, Italy) at 700–800 bar for 3 min. Then, the lysate was centrifuged for 30 min at 4 °C and 12,000 rpm, and the supernatants were loaded onto a Ni–NTA column. The N-terminal His-tagged *Pb*Ago and *Bl*Ago were eluted with elution buffer (20 mM Tris–HCl, 500 mM NaCl, 250 mM imidazole, 5 mM thioglycol, and pH 8.0). Finally, the purified protein was loaded onto a PD-10 desalting column (Sephadex G-25, GE Healthcare) and eluted with desalting buffer (20 mM Tris–HCl, 500 mM NaCl, 2 mM DTT, and pH 8.0). The eluted recombinant proteins were detected and analyzed by 15% SDS-PAGE. The concentrations of purified *Pb*Ago and *Bl*Ago were measured using a Nano-300 Micro-Spectrophotometer (Allsheng, China), and the fractions containing the protein were frozen at − 80 °C in storage buffer (20 mM Tris–HCl, 500 mM NaCl, 10% (v/v) glycerol, 2 mM DTT, and pH 8.0).

The purified Agos with 5 mM CaCl_2_ and 250 μg/mL proteinase K were incubated at 65 °C for 4 h. The bound nucleic acids were extracted from proteins by adding Roti phenol/chloroform/isoamyl alcohol pH 8.0 in a 1:1 ratio, and further precipitated by ethanol overnight at − 20 °C. The purified nucleic acids were treated with either RNase A or DNase I for 1 h at 37 °C, then resolved on 16% denaturing polyacrylamide gels, and stained with SYBR gold.

### Enzymatic characteristics of *Pb*Ago and *Bl*Ago in vitro

#### Single-stranded activity assays

The 5′-phosphorylated (5′-P) and 5′-hydroxylated (5′-OH) ssDNA or ssRNA guides and fluorescently labeled ssDNA or ssRNA targets were synthesized commercially (GenScript, China). For activity assays, 3 μM *Pb*Ago/1.5 μM *Bl*Ago, 0.5 μM ssDNA or ssRNA guide, and 0.1 μM fluorescently labeled ssDNA or ssRNA target were mixed in a reaction buffer (15 mM Tris–HCl, 200 mM NaCl, 0.5 mM MnCl_2_, and pH 8.0). The target was added after Ago and guide was incubated for 15 min at 37 °C. Then, the reaction mixture was incubated for 30 min at 37 °C. The reactions were stopped by the addition of loading buffer (95% formamide, 0.5 mM EDTA, 0.025% bromophenol blue, and 0.025% xylene cyanol FF) at a 1:1 ratio (v/v). Then, the samples were resolved on 16% denaturing polyacrylamide gels, stained with SYBR Gold (Invitrogen), and visualized using a Gel Image System (Tanon-3500BR).

#### *Pb*Ago and *Bl*Ago activity at varying temperatures

The reaction system was kept unchanged for some time. After, the ssDNA target was added and the complete reaction mixture was incubated at 10 °C, 20 °C, 30 °C, 37 °C, 45 °C, 55 °C, 65 °C, 75 °C, 85 °C, and 95 °C, for 30 min with 5′-P guide DNA (gDNA) and 2 h with 5′-OH gDNA, respectively. The samples were resolved on 16% denaturing polyacrylamide gels, stained with SYBR Gold, visualized with a Gel Image System (Tanon-3500BR), and analyzed using ImageJ and GraphPad Prism software (version 8.0).

#### Effect of divalent cations on *Pb*Ago and *Bl*Ago activity

For the assays, 0.5 mM of different divalent metal ions (MgCl_2_, MnCl_2_, FeCl_2_, CoCl_2_, CuCl_2_, NiCl_2_, ZnCl_2_, and CaCl_2_) was added to the reaction system, keeping other ingredients unchanged. The complete reaction mixture was then incubated for 30 min with 5′-P gDNA and 2 h with 5′-OH gDNA at 37 °C/65 °C, respectively. Cleavage activity without the addition of a divalent cation was used as a control.

The optimal Mn^2+^ concentration for *Pb*Ago and *Bl*Ago cleavage activity was also determined using buffers with different final concentrations of Mn^2+^: 5, 10, 25, 50, 100, 250, 500, 1000, 2000, and 3000 μM. All samples were stained and analyzed as described above.

#### Effect of NaCl concentration on *Pb*Ago and *Bl*Ago activity

The effect of NaCl concentration on the catalytic activity of *Pb*Ago and *Bl*Ago was investigated using reaction buffer systems with various NaCl concentrations (50, 100, 250, 500, 750, 1000, 1500, 2000, 2500, and 3000 mM). The samples were stained and analyzed as described above.

### Kinetic performance of *Pb*Ago and *Bl*Ago mediated by different guides

For cleavage kinetic analysis, the concentrations of *Pb*Ago/*Bl*Ago, ssDNA targets, and different guides were the same as shown above. The assays were performed with 50 mM NaCl and 2 mM Mn^2+^ at 37 °C/65 °C for different times: 0, 3, 5, 10, 20, 30, 45, 60, 80, 100, 120, 150, and 180 min. The samples were stained and analyzed as described above.

#### Effect of the length of 5′-P gDNA on *Pb*Ago and *Bl*Ago activity

Different lengths of the 5′-P gDNA that are complementary to the fluorescently labeled 78 nt ssDNA target were designed and synthesized. The other components in the reaction system and the reaction conditions were unchanged. Next, 0.5 μM 5′-P gDNA of different lengths was added to the system and the reaction was carried out at 37 °C/65 °C for 30 min. Samples without gDNA were used as the control group. The samples were stained and analyzed as described above.

#### Effect of the 5′-terminal nucleotide of gDNA on *Pb*Ago and *Bl*Ago activity

gDNAs with different 5′-terminal nucleotides complementary to the fluorescently labeled ssDNA targets were synthesized. The other components in the reaction system and the reaction conditions were unchanged. Next, 0.5 μM gDNA with different 5′-terminal nucleotides was added to the system and the reaction was carried out at 37 °C/65 °C for 30 min with the 5′-P gDNA and 2 h with the 5′-OH gDNA, respectively. Samples without gDNA were used as the control group. The samples were stained and analyzed as described above.

#### Effect of single-nucleotide mismatch on *Pb*Ago and *Bl*Ago activity

The 5′-P gDNAs with a single-nucleotide mismatch at different positions were synthesized. The other components in the reaction system and the reaction conditions were unchanged. Next, 0.5 μM 5′-P gDNA with single-nucleotide mismatch to the system was added and the reaction was carried out at 37 °C/65 °C for 30 min. Samples without gDNA were used as the control group. The samples were stained and analyzed as described above.

#### Effect of dinucleotide mismatches on *Pb*Ago and *Bl*Ago activity

The 5′-P gDNAs with dinucleotide mismatches at different positions were synthesized. The other components in the reaction system and the reaction conditions were unchanged. Next, 0.5 μM 5′-P gDNA with dinucleotide mismatches to the system was added and the reaction was carried out at 37 °C/65 °C for 30 min. Samples without gDNA were used as the control group. The samples were stained and analyzed as described above.

### Double-stranded activity assays

A pair of 5′-P and 5′-OH gDNAs complementary to the target fragment of pUC19 was synthesized. *Pb*Ago (3 μM)/*Bl*Ago (1.5 μM) and a pair of 0.5 μM gDNAs was mixed in the reaction buffer (15 mM Tris–HCl, 50 mM NaCl, 2 mM MnCl_2_, and pH 8.0) and incubated for 15 min at 37 °C. Then, 600 ng of pUC19 plasmid was added, after which the mixture was incubated for 3 h at 37 °C/65 °C. The reactions were stopped by treatment with Proteinase K at 4 °C, and the samples were mixed with 5 × loading buffer (Generay) and the cleavage products were resolved using 1% agarose gel electrophoresis.

### Kinetic performance of *Pb*Ago- and *Bl*Ago-cleaving plasmids

The reaction system remained unchanged, and then the reaction mixture was incubated at 37 °C /65 °C for different times: 0 h, 0.5 h, 1 h, 1.5 h, 2 h, 2.5 h, 3 h, 4 h, 5 h, 6 h, and 16 h, measuring the kinetic performance of *Pb*Ago- and *Bl*Ago-cleaving plasmids with 5′-P and 5′-OH gDNAs. The reactions were stopped and analyzed as described above.

#### Effect of the GC content of plasmid target fragment on *Pb*Ago and *Bl*Ago cleavage activity

The concentration of *Pb*Ago/*Bl*Ago and plasmid remained unchanged, and a pair of gDNAs complementary to fragments with different GC contents was mixed in the reaction buffer (15 mM Tris–HCl, 50 mM NaCl, 2 mM MnCl_2_, and pH 8.0) and reacted for 3 h at 37 °C /65 °C. The reactions were stopped and analyzed as described above.

#### Detection of small molecule by *Pb*Ago and *Bl*Ago with allosteric transcription factor HosA

The recombinant plasmid pET-21b (+)-HosA, containing the synthesized codon-optimized HosA gene, was constructed (Genscript, China) and transformed into the *E. coli* BL21 (DE3) strain to express and purify HosA.

60 nt ssDNA and 26 nt ssDNA containing the HosA recognition sequence were synthesized and annealed to form irregular dsDNA. Next, 0.1 μM of the irregular dsDNA was incubated for 30 min at 30 °C with different concentrations (0, 0.2, 0.3, 0.4, and 0.5 μM) of purified HosA in binding buffer (10 mM Tris–HCl, 100 mM KCl, 1 mM EDTA, 0.1 mM DTT, 5% (v/v) glycerol, 0.01 mg/mL bovine serum albumin, and pH 7.5) (Hellman and Fried 2007), to verify the combination of HosA with irregular dsDNA. For the dissociation assays, different concentrations of the p-HBA were added to the binding reaction mixtures. After incubation at 30 °C for 30 min, the samples were mixed with 10 × loading buffer (50% [v/v] glycerol and 0.1% [w/v] bromophenol blue) and resolved by 8% native PAGE with 1 × Tris–borate-EDTA buffer. Nucleic acids were visualized using a gel image system (Tanon-3500BR).

For detection of p-HBA, irregular dsDNA and HosA were added to the reaction system and incubated at 30 °C for 30 min. Next, the target small molecule p-HBA was added to the reaction system, inducing the dissociation of HosA–dsDNA under the same conditions. Finally, Ago/5′-P gDNA was added to cleave the free irregular dsDNA at 30 °C for 30 min. The samples were resolved on 16% denaturing polyacrylamide gels, stained, and analyzed as described above.

## Results and discussion

### *Pb*Ago and *Bl*Ago utilize both 5′-P and 5′-OH DNA guides for target cleavage

**Fig. 1:**
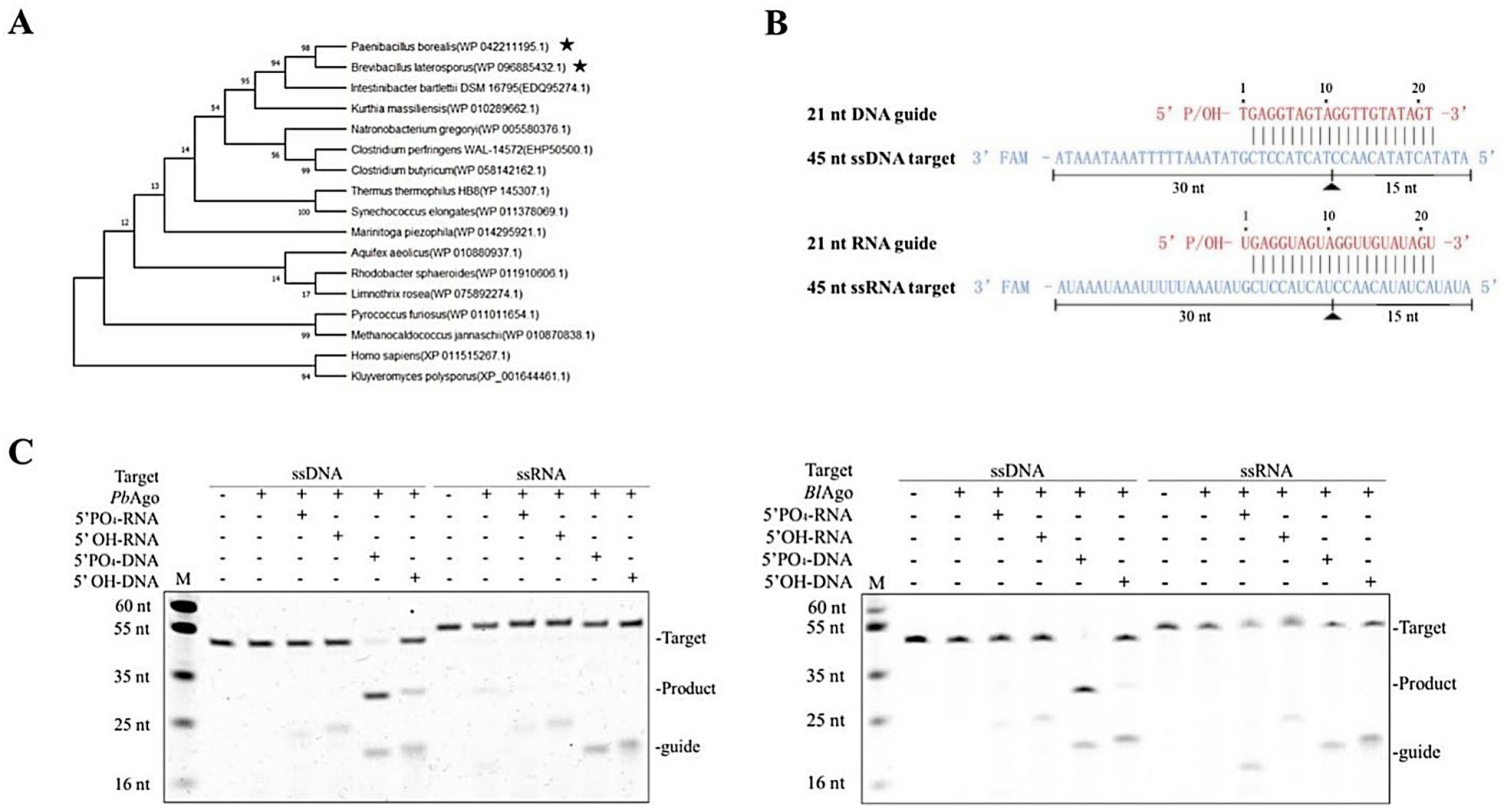
*Pb*Ago and *Bl*Ago exhibit DNA-guided DNA cleavage activity at 37 °C. **a** Neighbor-joining phylogenetic tree analysis of *Pb*Ago and *Bl*Ago and characterized pAgos. The numbers at the nodes indicate the bootstrap values for neighbor-joining analysis of 1000 resampled data sets. The scale bar represents the evolutionary distance between species. The numbers in parentheses represent the sequence accession numbers in the NCBI database. **b** Synthetic DNA or RNA guides (red) and targets (blue). The predicted cleavage positions are indicated with a black triangle; black lines indicate the predicted 15 and 30 nt cleavage products. **c** Cleavage activity assays of *Pb*Ago and *Bl*Ago with FAM-labeled DNA or RNA targets

*Pb*Ago and *Bl*Ago were chosen as candidates because they were phylogenetically closest to mesophilic *Ib*Ago (Fig. 1a). The sequence identity of *Pb*Ago and *Ib*Ago was 35%, while *Bl*Ago shared 39.4% identity with *Ib*Ago. *Ib*Ago has been reported to cleave target DNA in an ssDNA-dependent manner. It has been reported that targeted cleavage of all catalytically active Agos is mediated by a conserved DEDX catalytic residues (where X can be D, H, N, or K) (Sheng et al. 2014; Swarts et al. 2015, 2014b; Willkomm et al. 2017). The multiple sequence alignment result showed that *Pb*Ago and *Bl*Ago contain the DEDD tetrad (Additional file 1: Fig. S1a), suggesting that *Pb*Ago and *Bl*Ago may have endonuclease catalytic activity that needs to be characterized in vitro.

The genes encoding *Pb*Ago and *Bl*Ago were cloned into the plasmid pET-28a(+)-TEV and then successfully expressed in *E. coli* BL21 (DE3). SDS-PAGE analysis showed that the size of the purified proteins was consistent with the estimated molecular weight of the recombinant *Pb*Ago (81 kDa) and *Bl*Ago (80 kDa) (Additional file 1: Fig. S1b). We isolated the nucleic acid fraction that co-purified with *Pb*Ago and *Bl*Ago, and verified that both bound ~ 16 nucleotide long siDNAs in vivo (Additional file 1: Fig. S2).

**Fig. 2:**
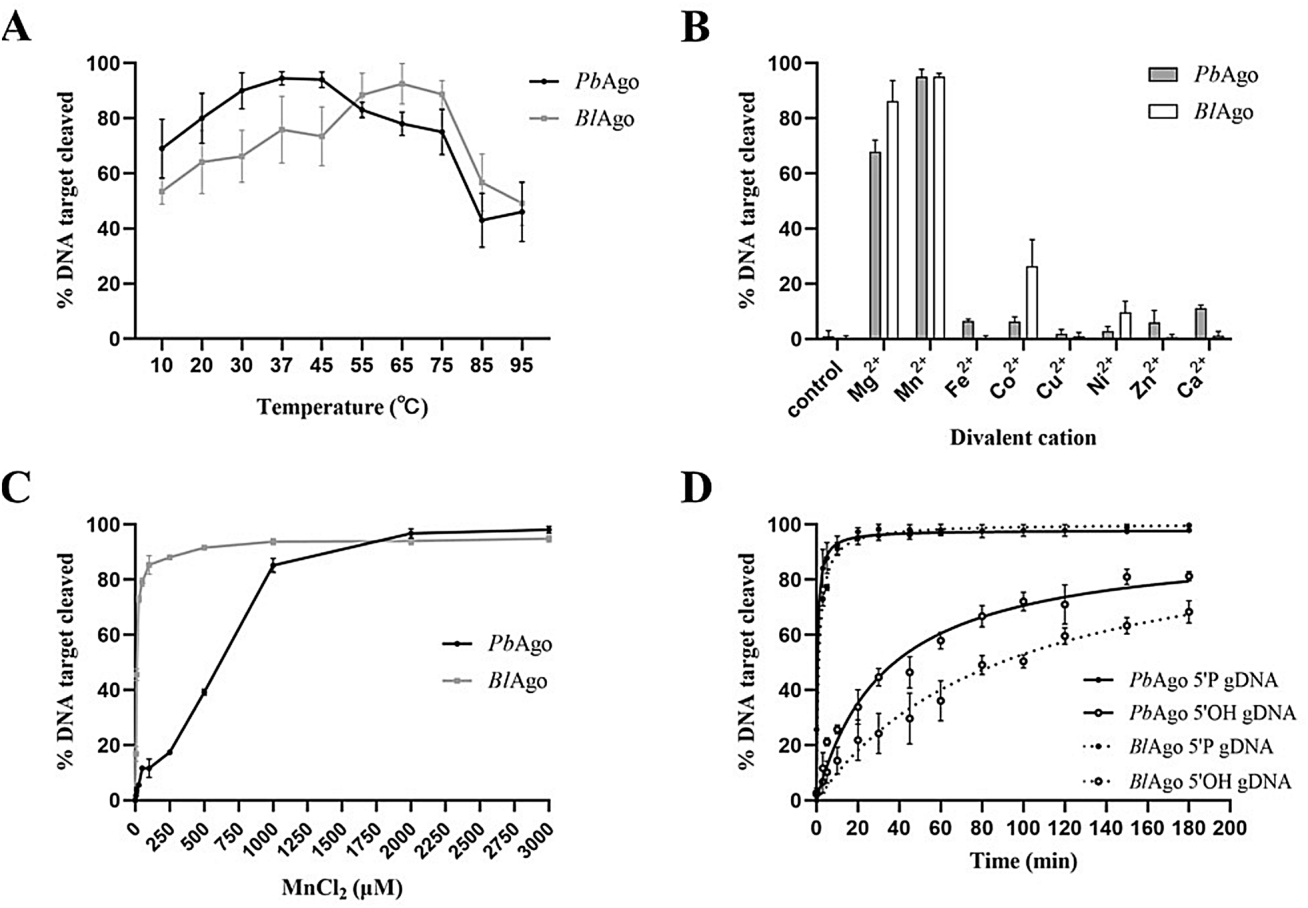
Enzymatic characterization of *Pb*Ago and *Bl*Ago in vitro guided by the 5′-P ssDNA. **a** Effect of temperature on *Pb*Ago and *Bl*Ago activity mediated by the 5′-P gDNA. **b** Effects of different divalent cations on *Pb*Ago and *Bl*Ago activity mediated by the 5′-P gDNA. **c** Effects of Mn^2+^ concentrations on *Pb*Ago and *Bl*Ago activity mediated by the 5′-P gDNA. **d** Kinetic performance of *Pb*Ago and *Bl*Ago mediated by 5′-P and 5′-OH gDNAs. Error bars represent the SDs of three independent experiments

To study the enzymatic properties of *Pb*Ago and *Bl*Ago, we investigated nucleic acid cleavage through various nucleic acid guides in vitro. First, 21-nucleotide (nt) ssDNA or ssRNA guides with a 5′-Phosphate or 5′-hydroxyl group were designed to targeting 45 nt 3′6-FAM-labeled ssDNA or ssRNA targets (Fig. 1b). The results of cleavage assays showed that both *Pb*Ago and *Bl*Ago use 5′-P gDNA or 5′-OH gDNA to cleave complementary ssDNA targets (Fig. 1c). However, the cleaving efficiencies of *Pb*Ago and *Bl*Ago mediated by 5′-P gDNA were higher than that of 5′-OH gDNA (Fig. 2a and Additional file 1: Fig. S3a).

### Requirements for target cleavage by *Pb*Ago and *Bl*Ago

To further investigate the full temperature range at which *Pb*Ago and *Bl*Ago are active, we performed cleavage assays at temperatures ranging from 10 to 95 °C. The optimum temperatures of *Pb*Ago were significantly different when directed by various guides: the 5′-P ssDNA guide-mediated *Pb*Ago was most active at 37 °C (Fig. 2a); *Pb*Ago displayed the highest activity at 65 °C, directed by the 5′-OH gDNA (Additional file 1: Fig. S3a); and *Bl*Ago displayed the highest activity at 65 °C, directed by both 5′-P and 5′-OH gDNAs (Fig. 2a and Additional file 1: Fig. S3a).

Agos are divalent cation-dependent endonucleases (Ji-Joon Song 2004; Nowotny et al. 2005), and the presence of divalent cations is essential for pAgos to specifically bind to the 5′end of the guide strand (Sheng et al. 2014; Wang et al. 2009). To study the preference of *Pb*Ago and *Bl*Ago for divalent cations, different divalent cations (Mg^2+^, Mn^2+^, Fe^2+^, Co^2+^, Cu^2+^, Ni^2+^, Zn^2+^, and Ca^2+^) were added to the reaction system. The results showed that *Pb*Ago was active with Mg^2+^ and Mn^2+^ directed by the 5′-P gDNA, while only Mn^2+^ promoted the cleavage activity of *Pb*Ago guided by 5′-OH gDNA (Fig. 2b and Additional file 1: Fig. S3b). In addition, the improvement of Mg^2+^ and Mn^2+^ on *Pb*Ago cleavage activity was quite different, directed by 5′-P and 5′-OH gDNAs. *Bl*Ago was active with Mg^2+^, Mn^2+^, Co^2+^, and Ni^2+^ directed by 5′-P gDNA, while Mn^2+^ and Co^2+^ promoted the cleavage activity of *Bl*Ago guided with 5′-OH gDNA; Mn^2+^ gave the best promotion (Fig. 2b and Additional file 1: Fig. S3b).

MnCl_2_ concentrations ranging from 250 to 3000 μM were used to further explore the optimal concentration of Mn^2+^ for the cleavage activity of *Pb*Ago and *Bl*Ago. Their cleavage activity mediated by 5′-P and 5′-OH gDNAs both increased with the increase in Mn^2+^ concentration, the optimal Mn^2+^ concentration for them depending on the guide. *Pb*Ago cleaved 90% of the target DNA directed by 5′-P gDNA with not less than 500 μM Mn^2+^, while *Pb*Ago directed by 5′-OH gDNA displayed the highest catalytic activity with not less than 2 mM Mn^2+^ (Fig. 2c and Additional file 1: Fig. S3c). *Bl*Ago cleaved 90% of the target DNA directed by 5′-P gDNA with not less than 100 μM Mn^2+^, while *Pb*Ago directed by 5′-OH gDNA displayed the highest catalytic activity with not less than 3 mM Mn^2+^ (Fig. 2c and Additional file 1: Fig. S3c).

NaCl plays an important role in the catalytic activity of Ago and the maintenance of enzyme stability (Swarts et al. 2014a, 2015). Therefore, we explored the effect of NaCl concentration on the cleavage activity of *Pb*Ago and *Bl*Ago mediated by 5′-P and 5′-OH gDNAs. It was found that NaCl inhibited the cleavage activity of *Pb*Ago directed by both 5′-P and 5′-OH gDNAs. *Pb*Ago displayed the highest catalytic activity with 50 mM NaCl (Additional file 1: Fig. S3d). With the gradual increase in NaCl concentration, the cleavage efficiency of *Bl*Ago first increased and then decreased; *Bl*Ago displayed the highest catalytic activity with 500 mM NaCl (Additional file 1: Fig. S3d).

To further investigate the catalytic properties of *Pb*Ago directed by 5′-P and 5′-OH gDNAs, we performed a cleavage kinetics assay at 37 °C/65 °C with 50 mM NaCl and 2 mM Mn^2+^. The results showed that the reaction rate of *Pb*Ago mediated by the 5′-P gDNA was faster than that mediated by the 5′-OH gDNA, and *Pb*Ago exhibited the highest cleavage efficiency guided by the 5′-P gDNA (Fig. 2d), indicating that *Pb*Ago prefers the 5′-P ssDNA as a guide. For *Bl*Ago, we performed a cleavage kinetics assay at 65 °C with 50 mM NaCl and 2 mM Mn^2+^. The results showed that the reaction rate of *Bl*Ago mediated by the 5′-P gDNA was faster than that mediated by the 5′-OH gDNA, and *Bl*Ago exhibited the highest cleavage efficiency guided by the 5′-P gDNA (Fig. 2d), indicating that *Bl*Ago prefers the 5′-P ssDNA as a guide.

### The sequence of the nucleic acid guide affects *Pb*Ago and *Bl*Ago activity

**Fig. 3:**
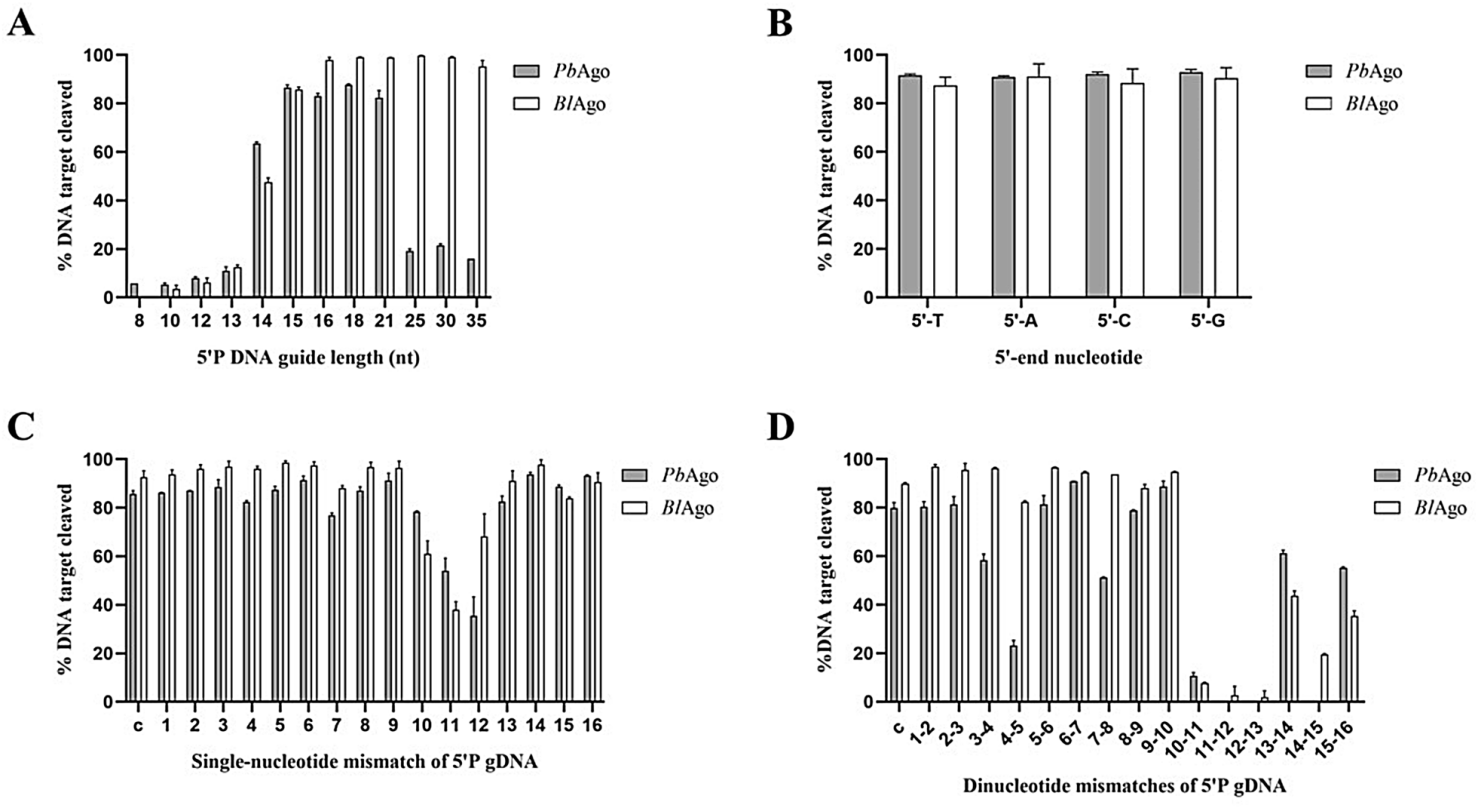
Effect of guide sequence on *Pb*Ago and *Bl*Ago. **a** Effect of guide length on *Pb*Ago and *Bl*Ago. **b** Effect of the 5′-terminal nucleotide of the 5′-P gDNA on *Pb*Ago and *Bl*Ago. **c** Target ssDNA cleavage activity of *Pb*Ago and *Bl*Ago loaded with single-nucleotide mismatched gDNA. **d** Effect of dinucleotide mismatches on *Pb*Ago and *Bl*Ago activity. Error bars represent the SDs of three independent experiments

The length of the guide has been reported to affect the cleavage efficiency of pAgos (Hegge et al. 2019; Kuzmenko et al. 2019; Liu et al. 2021b). We added 5′-P gDNA of different lengths to the reaction system and investigated the effect of guide length on the cleavage efficiency of *Pb*Ago and *Bl*Ago. The results showed that the cleavage efficiency of *Pb*Ago remained at a high level when gDNA was in the range of 15–21 nt. As the length of gDNA increased, the cleavage efficiency of *Pb*Ago decreased (Fig. 3a). When the gDNA was in the range of 15–35 nt, the cleavage efficiency of *Bl*Ago remained high. *Bl*Ago displayed the highest catalytic activity with 16 nt 5′-P gDNA (Fig. 3a).

To investigate whether the 5′-terminal nucleotide of the gDNA affects the activity of *Pb*Ago and *Bl*Ago, we performed cleavage assays. Four types of gDNA with different 5′-terminal nucleotides (A/T/C/G) were incubated with *Pb*Ago and *Bl*Ago, and then incubated with complementary target DNA. The cleavage efficiency of *Pb*Ago was essentially the same and mediated by 5′-P gDNA with different 5′-terminal nucleotides. Directed by 5′-OH gDNA with different 5′-terminal nucleotides, the cleavage efficiency of *Pb*Ago was slightly lower with DNA guides containing a 5′-A (Fig. 3b and Additional file 1: Fig. S4). The cleavage efficiency of *Bl*Ago was essentially the same and mediated by 5′-P gDNA with different 5′-terminal nucleotides (A/T/C/G). Directed by 5′-OH gDNA with different 5′-terminal nucleotides (A/T/C/G), the cleavage efficiency of *Bl*Ago was significantly reduced with gDNA containing a 5′-T or 5′-A; *Bl*Ago displayed the lowest cleavage efficiency directed by gDNA containing a 5′-A (Fig. 3b and Additional file 1: Fig. S4).

Previous studies on eAgos and several pAgos, including *Af*Ago (*Archaeoglobus fulgidus*) (Parker et al. 2009), *Tt*Ago, *Rs*Ago (*Rhodobacter sphaeroides*) (Olovnikov et al. 2013), *Mp*Ago (*Marinitoga piezophila*) (Kaya et al. 2016), *Cb*Ago, *Lr*Ago, and *Km*Ago, showed that mismatches between the guide and the target may have significant effects on target recognition and cleavage efficiency. We first introduced a single mismatched nucleotide at different positions of gDNA to verify the effect of a single mismatch on the cleavage activity of *Pb*Ago and *Bl*Ago. We observed that the introduction of single mismatches at positions 10–12 of gDNA inhibited the cleavage of *Pb*Ago and *Bl*Ago more significantly. This may be because positions 10 and 11 of gDNA are the key sites for Ago to perform cleavage activity (Fig. 3c). Because *Pb*Ago and *Bl*Ago were more resistant to single mismatch introduced into gDNA, to further explore their mismatch tolerance and splicing specificity, we introduced consecutive dinucleotide mismatches at positions 1–16 of the gDNA. We found that after introducing dinucleotide mismatches at positions 3–4, 7–8, 13–14, and 15–16, the cutting efficiency of *Pb*Ago decreased by 20–30%; after introducing dinucleotide mismatches at positions 4–5, 10–13, and 14–15, the cleavage efficiency of *Pb*Ago dropped sharply to 18–25%, and even completely lost the cleavage activity (Fig. 3c). The introduction of dinucleotide mismatches at positions 4–5 had a weaker effect on the cleavage efficiency of *Bl*Ago; after the introduction of dinucleotide mismatches at positions 10–13, the cutting efficiency of *Bl*Ago almost dropped to 0%, and after introducing dinucleotide mismatches at positions 13–16, the cleavage efficiency of *Bl*Ago gradually increased to 20–45% (Fig. 3d).

### Cleavage of double-stranded DNA by *Pb*Ago and *Bl*Ago

**Fig. 4:**
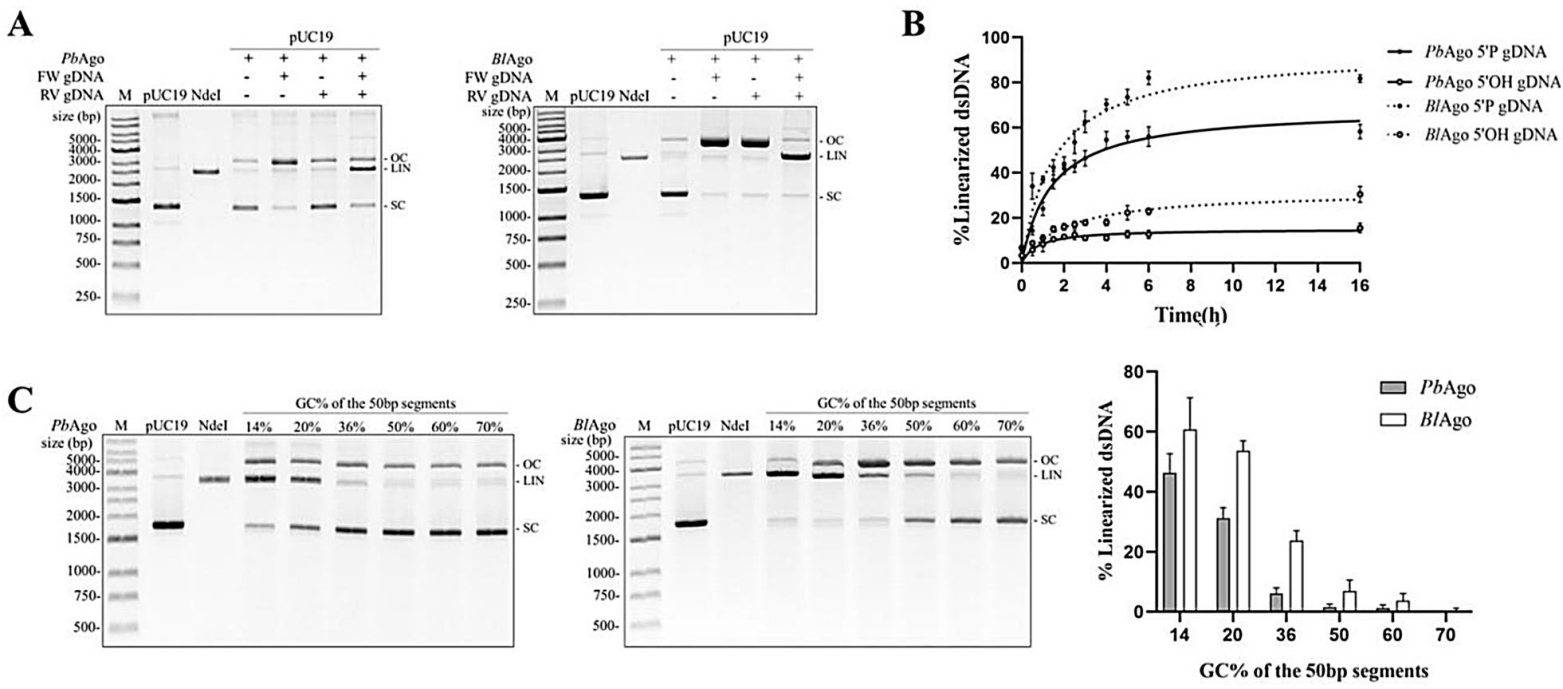
Cleavage of pUC19 by *Pb*Ago and *Bl*Ago. **a** *Pb*Ago cleaves pUC19 at 37 °C guided by a pair of 5′-P gDNAs. *Bl*Ago cleaves pUC19 at 65 °C guided by a pair of 5′-P gDNAs. **n** Kinetic performance of *Pb*Ago- and *Bl*Ago-cleaving pUC19. **c** Effect of GC content of 50 bp plasmid target fragment on *Pb*Ago and *Bl*Ago cleavage activity. FW/RV gDNA, forward and reverse gDNAs; M, molecular weight marker; OC, open circular plasmid; Lin, linearized plasmid; SC, supercoiled plasmid

Since pAgo-gDNA complex can only bind and cleave ssDNA, two individual pAgo-gDNA complexes were needed to make dsDNA break, each targeting one strand of the target dsDNA. Although all pAgos characterized to date seem to lack the ability to actively unwind dsDNA, it has been reported that thermophilic pAgos can be used to generate dsDNA breaks in plasmid DNA in vitro (Ketting 2011; Pratt and MacRae 2009). Thermophilic pAgos rely on elevated temperatures (≥ 65 °C) to promote local unwinding, and then two pAgo-gDNA complexes target two strands of DNA separately. However, both *Pb*Ago and *Bl*Ago are derived from mesophilic organisms. To test whether they can cleave dsDNA substrates at moderate temperatures, we incubated apo-Ago and pre-assembled Ago-gDNA complexes with the target plasmid (pUC19) at 37 °C and 65 °C. Previous studies have shown that *Cb*Ago (Hegge et al. 2019; Kuzmenko et al. 2019), *Lr*Ago (Kuzmenko et al. 2019), and *Km*Ago (Liu et al. 2021b) can relax supercoiled plasmid DNA in a guide-independent manner. Here, we also found that apo-*Pb*Ago and apo-*Bl*Ago could nick the supercoiled plasmid substrate, converting it from supercoiled state to open circular state (Fig. 4a). When the plasmid was targeted by *Pb*Ago/*Bl*Ago with a single gDNA, we also observed a reduction in supercoiled plasmids and accumulation of open circular plasmids (Fig. 4a). When adding a pair of gDNAs, each *Pb*Ago/*Bl*Ago-gDNA complex targeting one strand of the plasmid, we observed a portion of the linearized plasmid DNA (Fig. 4a). These results implied that nicking of each strand of the target plasmid mediated by the *Pb*Ago/*Bl*Ago-gDNA complexes leads to double-stranded DNA breaks, and the cleavage efficiency of *Pb*Ago/*Bl*Ago directed by 5′-P gDNA was higher than that of 5′-OH gDNA (Additional file 1: Fig. S5).

Next, we measured the kinetics of *Pb*Ago- and *Bl*Ago*-*cleaving pUC19 mediated by 5′-P and 5′-OH gDNAs. The results showed that the reaction rates of *Pb*Ago and *Bl*Ago mediated by the 5′-P gDNA were faster than that mediated by the 5′-OH gDNA, and the cleavage efficiency mediated by the 5′-P gDNA was higher (Fig. 4b), which could cleave most of pUC19.

It has been reported that *Cb*Ago (Hegge et al. 2019; Kuzmenko et al. 2019), *Lr*Ago (Kuzmenko et al. 2019), *Km*Ago (Liu et al. 2021b), *Cp*Ago (Cao et al. 2019), and *Ib*Ago (Cao et al. 2019) could cleave AT-rich dsDNA more effectively than GC-rich dsDNA, probably because the AT-rich dsDNA was easier to unwind. To test whether *Pb*Ago and *Bl*Ago have this preference, we selected six target DNA fragments with different GC contents of 50 bp on plasmid pUC19, and then designed six pairs of gDNAs respectively. It was found that the lower the GC content of the 50 bp target DNA fragment, the better the cleavage efficiency of *Pb*Ago and *Bl*Ago (Fig. 4c). *Pb*Ago could cleave dsDNA fragments with a GC content of 36% or lower, and *Bl*Ago could cleave dsDNA fragments with a GC content of 50% or lower.

### Small molecule detection by *Pb*Ago and *Bl*Ago and allosteric transcription factors

**Fig. 5:**
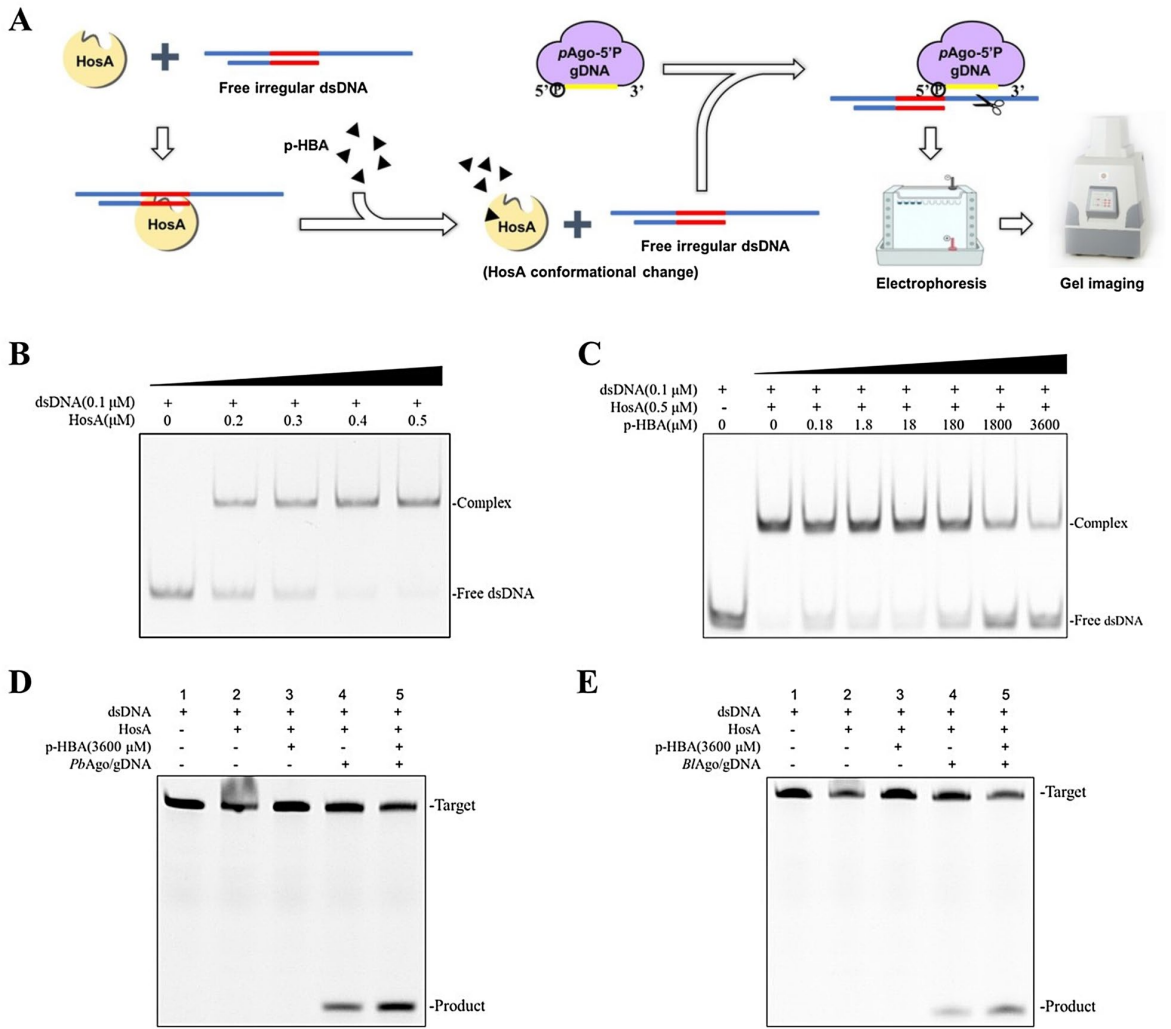
Small molecule detection by *Pb*Ago and *Bl*Ago and allosteric transcription factors. **a** Schematic diagram of small molecule detection by *Pb*Ago and *Bl*Ago and allosteric transcription factors. **b** EMSA with HosA and irregular dsDNA containing the HosA-binding motif. **c** EMSA evaluation of HosA allosteric activity in the presence of p-HBA. **d** The denaturing polyacrylamide gel electrophoresis (PAGE) result of *Pb*Ago detecting p-HBA. The denaturing PAGE with irregular dsDNA (lane 1), with irregular dsDNA incubated with HosA (lane 2), with irregular dsDNA incubated with HosA and p-HBA (lane 3), with irregular dsDNA incubated with HosA and *Pb*Ago/gDNA (lane 4), and with irregular dsDNA incubated with HosA, p-HBA, and *Pb*Ago/gDNA (lane 5). **e** The denaturing PAGE result of *Bl*Ago detected p-HBA. The denaturing PAGE with irregular dsDNA (lane 1), with irregular dsDNA incubated with HosA (lane 2), with irregular dsDNA incubated with HosA and p-HBA (lane 3), with irregular dsDNA incubated with HosA and *Bl*Ago/gDNA (lane 4), and with irregular dsDNA incubated with HosA, p-HBA, and *Bl*Ago/gDNA (lane 5)

We also used *Pb*Ago and *Bl*Ago and the allosteric transcription factor HosA to detect the small molecule p-HBA (Fig. 5a). We first constructed the HosA gene into the pET-21b(+) vector, synthesized the pET-21b(+)-HosA plasmid (GenScript, China), and then transformed *E. coli* BL21 (DE3) to express and purify HosA in vitro (Additional file 1: Fig. S6). Since *Pb*Ago and *Bl*Ago cannot cleave linear dsDNA, two complementary ssDNAs with different lengths were synthesized, which contained a HosA-specific recognition sequence after annealed to an irregular dsDNA (Fig. 5a). Electrophoretic mobility shift assay (EMSA) with HosA and irregular dsDNA containing the HosA-binding motif showed that the irregular dsDNA could be bound by HosA (Fig. 5b). In our system, the dsDNA substrate could be released from HosA in the presence of p-HBA (Fig. 5c). A 5′-P gDNA complementary to the overhang of the irregular dsDNA was added to confirm whether Ago/gDNA complex could cleave the overhang of the irregular dsDNA and produce an ssDNA product. The ssDNA could easily be detected which illustrated the presence of small molecule p-HBA.

To this end, Ago/gDNA and Ago/gDNA/p-HBA were added to the HosA–dsDNA complex (Fig. 5d, e). The results showed that more cleavage products could be detected in the group contain p-HBA compared with the group without p-HBA (Fig. 5d, e), demonstrated that *Pb*Ago and *Bl*Ago cleaved released irregular dsDNA after p-HBA bound to HosA. The group without p-HBA also existed cleavage products, probably because remanent free irregular dsDNA not bound by HosA in the reaction system could be cleaved by Ago/gDNA. Another possible reason was that Ago/gDNA competed with HosA to bind to dsDNA. Compared to CRISPR-Cas detection system (Li et al. 2018), our system possesses the following advantages. Firstly, there is no requirement of specific protospacer-adjacent motifs (PAM) for the targets. Secondly, the synthetic DNA guides show lower cost and higher stability than crRNAs used in CRISPR-Cas-based assays. In the future, we shall improve the readout system for rapid and easy detection by combining fluorescent reporters.

## Conclusion

In this study, we presented a detailed characterization of pAgos from the mesophilic bacteria *Paenibacillus borealis* and *Brevibacillus laterosporus*. We demonstrated that *Pb*Ago and *Bl*Ago could utilize both 5′-P and 5′-OH ssDNA guides to cleave target DNA substrates at moderate temperatures. *Pb*Ago displayed the highest cleavage activity with 50 mM NaCl, whereas *Bl*Ago displayed the highest cleavage activity with 500 mM NaCl. For the influence of gDNA sequence on enzyme activity, the introduction of single and dinucleotide mismatches at positions 10–12 of gDNA will inhibit the cleavage of *Pb*Ago and *Bl*Ago more significantly.

We also observed that *Pb*Ago and *Bl*Ago could utilize a pair of both 5′-P and 5′-OH gDNAs to generate double-stranded DNA breaks in plasmid DNA. Both *Pb*Ago and *Bl*Ago could nick one strand of pUC19, converting it from supercoiled to open circular state. Characterization of *Pb*Ago and *Bl*Ago expands the understanding of the pAgo family and will inspire the development of the potential applications of these new Ago proteins in genome editing.

Detection of the small molecule p-HBA is of great significance for scientific research, food safety, and disease diagnosis. In this study, we developed a simple and low-cost p-HBA detection method based on DNA-guided DNA cleavage of *Pb*Ago/*Bl*Ago and the allosteric effect of HosA, which expanded the potential application of small molecule detection by pAgos.

### Supplementary Information

**Additional file 1: Fig. S1.** *Pb*Ago and *Bl*Ago contain the catalytic DEDD tetrad. **Fig. S2.** Nucleic acids that co-purified with *Pb*Ago and *Bl*Ago. **Fig. S3.** Enzymatic characterization of *Pb*Ago and *Bl*Ago in vitro guided by 5′-OH ssDNA. **Fig. S4.** Effect of 5′-terminal nucleotide of the 5′-OH ssDNA gDNA on *Pb*Ago and *Bl*Ago. **Fig. S5.** *Pb*Ago and *Bl*Ago cleaves pUC19 guided by 5’OH gDNA. **Fig. S6. **SDS-PAGE analysis of Ni–NTA-purified HosA. **Table S1.** Nucleic acids used in ssDNA cleavage. **Table S2.** Nucleic acids used in dsDNA cleavage. **Table S3.** Nucleic acids used in detection of p-HBA.

## Data Availability

All data generated or analyzed during this study are included in this published article.

## Abbreviations

Ago: Argonaute protein
aTF: Allosteric transcription factors
eAgo: Eukaryotic Argonaute protein
pAgo: Prokaryotic Argonaute protein
*Pb*Ago: *Paenibacillus borealis* Argonaute protein
*Bl*Ago: *Brevibacillus laterosporus* Argonaute protein
ssDNA: Single-stranded DNA
dsDNA: Double-stranded DNA
p-HBA: P-hydroxybenzoic acid
*Pf*Ago: *Pyrococcus furiosus* Argonaute protein
*Tt*Ago: *Thermus thermophilus* Argonaute protein
*Mj*Ago: *Methanocaldococcus jannaschii* Argonaute protein
*Cb*Ago: *Clostridium butyricum* Argonaute protein
*Lr*Ago: *Limnothrix rosea* Argonaute protein
*Cp*Ago: *Clostridium perfringen* Argonaute protein
*Ib*Ago: *Intestinibacter bartlettii* Argonaute protein
*Se*Ago: *Synechococcus elongatus* Argonaute protein
*Km*Ago: *Kurthia massiliensis* Argonaute protein
EBD: Effector binding domain
DBD: DNA binding domain
5′-P ssDNA: 5′-Phosphorylated single-stranded DNA
5′-OH ssDNA: 5′-Hydroxylated single-stranded DNA
*Af*Ago: *Archaeoglobus fulgidus* Argonaute protein
*Rs*Ago: *Rhodobacter sphaeroides* Argonaute protein
*Mp*Ago: *Marinitoga piezophila* Argonaute protein
