# Supplementary material for: Characterization of Argonaute nucleases from mesophilic bacteria Paenibacillus borealis and Brevibacillus laterosporus
Source: Bioresour Bioprocess. 2021 Dec 19;8(1):133. doi: 10.1186/s40643-021-00478-z (PMC10992608; doi:10.1186/s40643-021-00478-z)
Supplement: Supplementary file 1 — Additional file 1: Fig. S1. PbAgo and BlAgo contain the catalytic DEDD tetrad. Fig. S2. Nucleic acids that co-purified with PbAgo and BlAgo. Fig. S3. Enzymatic characterization of PbAgo and BlAgo in vitro guided by 5′-OH ssDNA. Fig. S4. Effect of 5′-terminal nucleotide of the 5′-OH ssDNA gDNA on PbAgo and BlAgo. Fig. S5. PbAgo and BlAgo cleaves pUC19 guided by 5’OH gDNA. Fig. S6. SDS-PAGE analysis of Ni–NTA-purified HosA. Table S1. Nucleic acids used in ssDNA cleavage. Table S2. Nucleic acids used in dsDNA cleavage. Table S3. Nucleic acids used in detection of p-HBA. [file 40643_2021_478_MOESM1_ESM.docx]

**Supplementary figures**


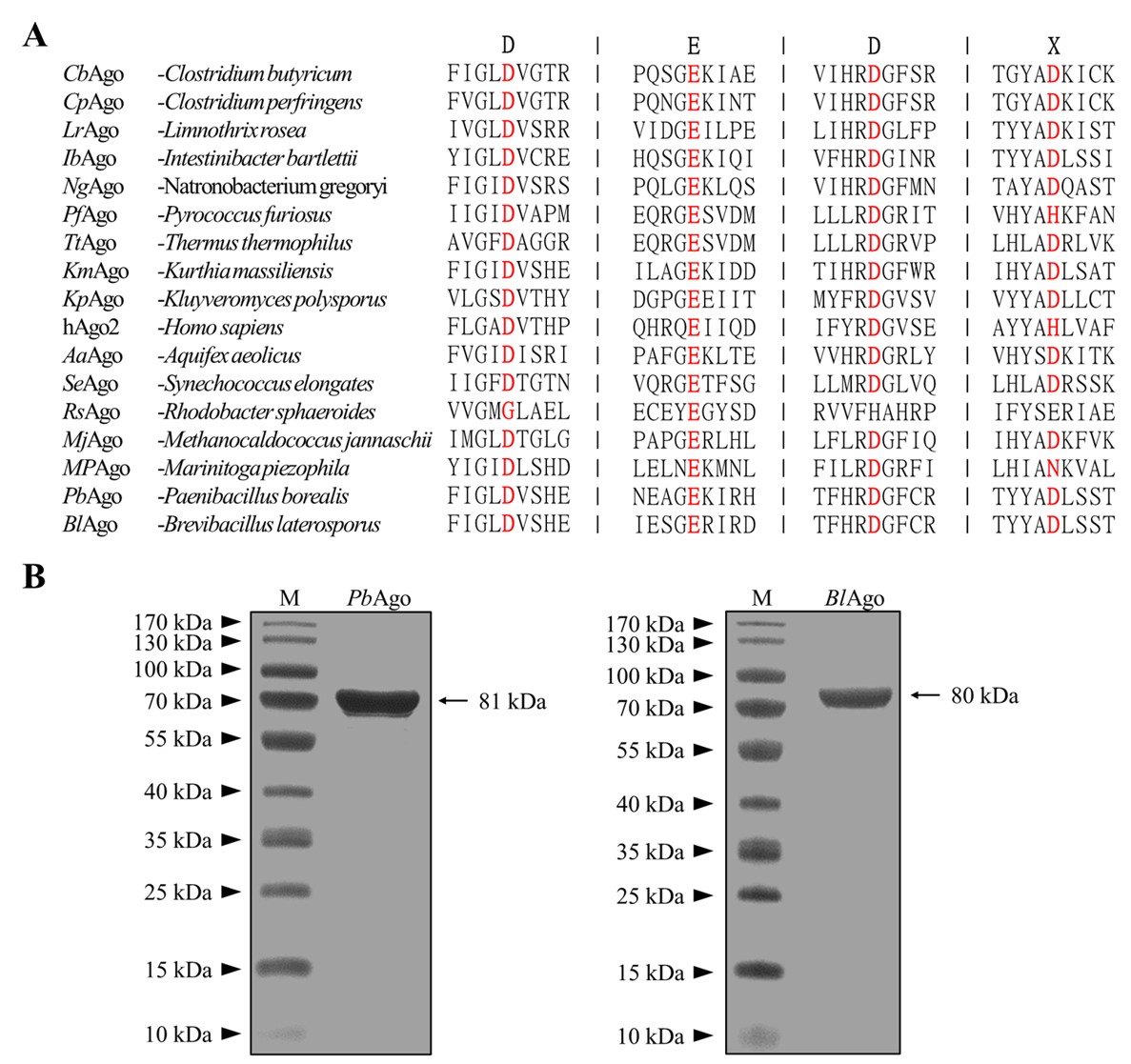


**Fig. S1.** *Pb*Ago and *Bl*Ago contain the catalytic DEDD tetrad. a) Multiple sequence alignment of the partial PIWI domain from *Pb*Ago, *Bl*Ago with other characterized pAgo proteins. b) SDS-PAGE analysis of Ni-NTA-purified *Pb*Ago and *Bl*Ago. M: protein marker.


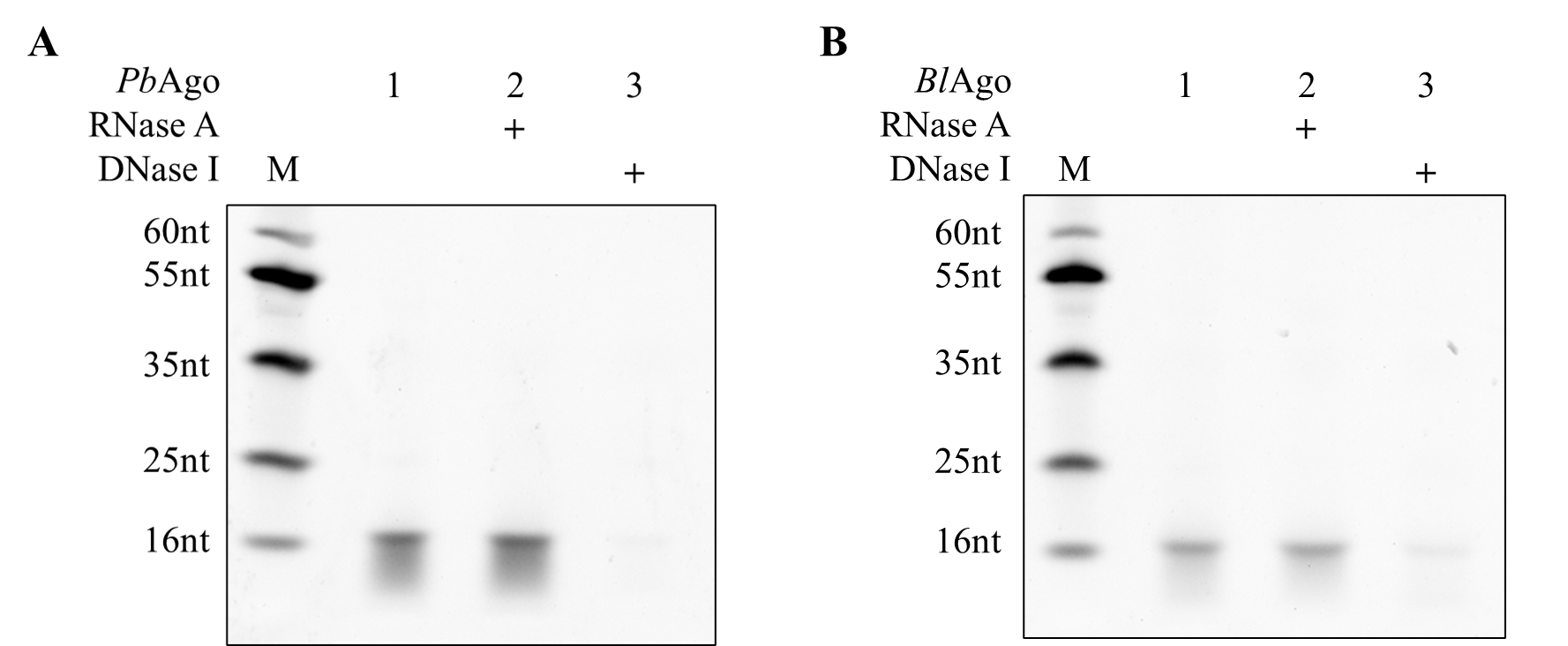


**Fig. S2.** Co-purified nucleic acids analysis of *Pb*Ago and *Bl*Ago. Nucleic acids that co-purified with *Pb*Ago and *Bl*Ago were isolated and treated with either RNAse A or DNAse I, then analyzed by denaturing polyacrylamide gel electrophoresis. *Pb*Ago and *Bl*Ago acquire 16 nucleotides long siDNAs *in vivo*.


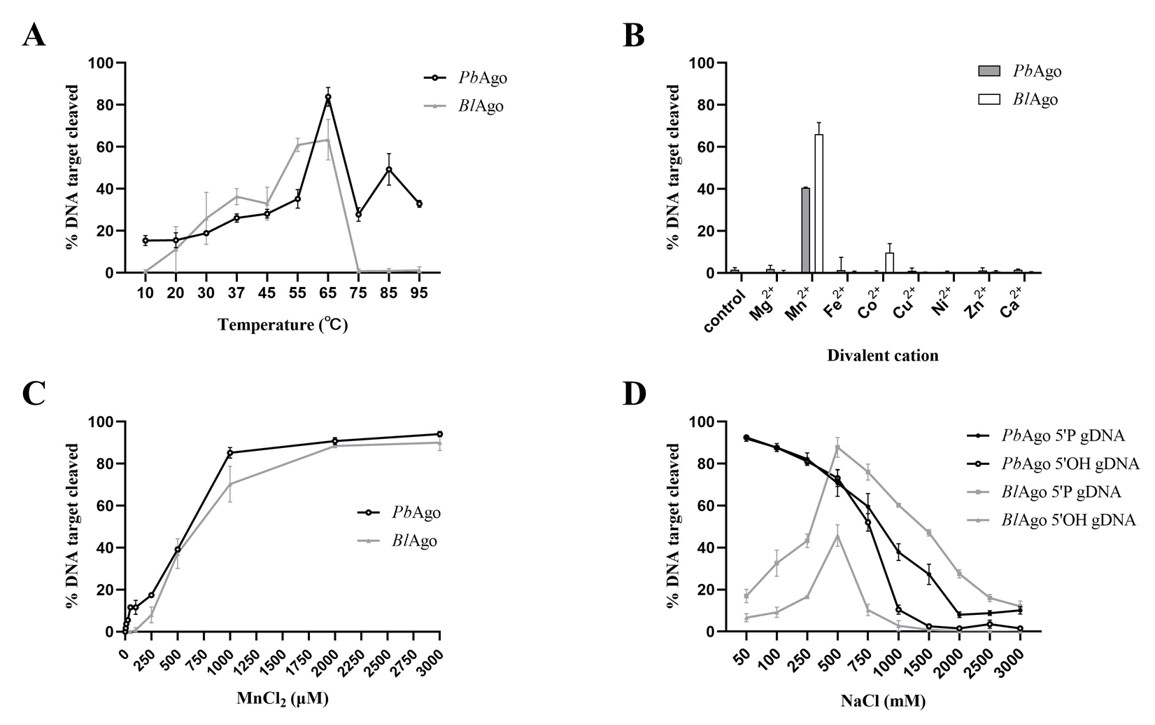


**Fig. S3.** Enzymatic characterization of *Pb*Ago and *Bl*Ago in vitro guided by 5’OH ssDNA. a) Effect of temperature on *Pb*Ago and *Bl*Ago activity mediated by 5’OH ssDNA guides. b) Effects of different divalent cations on *Pb*Ago and *Bl*Ago activity mediated by 5’OH ssDNA guides. c) Effects of Mn^2+^ concentrations on *Pb*Ago and *Bl*Ago activity mediated by 5’OH ssDNA guides. d) Effect of NaCl on *Bl*Ago activity mediated by 5’P/OH ssDNA guides. Error bars represent the SDs of three independent experiments.


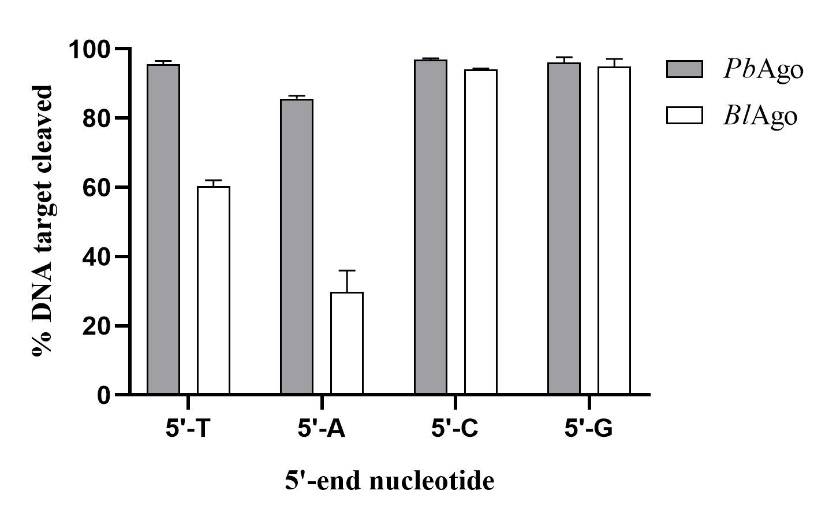


**Fig. S4.** Effect of 5’-terminal nucleotide of the 5’OH ssDNA guide DNA on *Pb*Ago and *Bl*Ago. Error bars represent the SDs of three independent experiments.


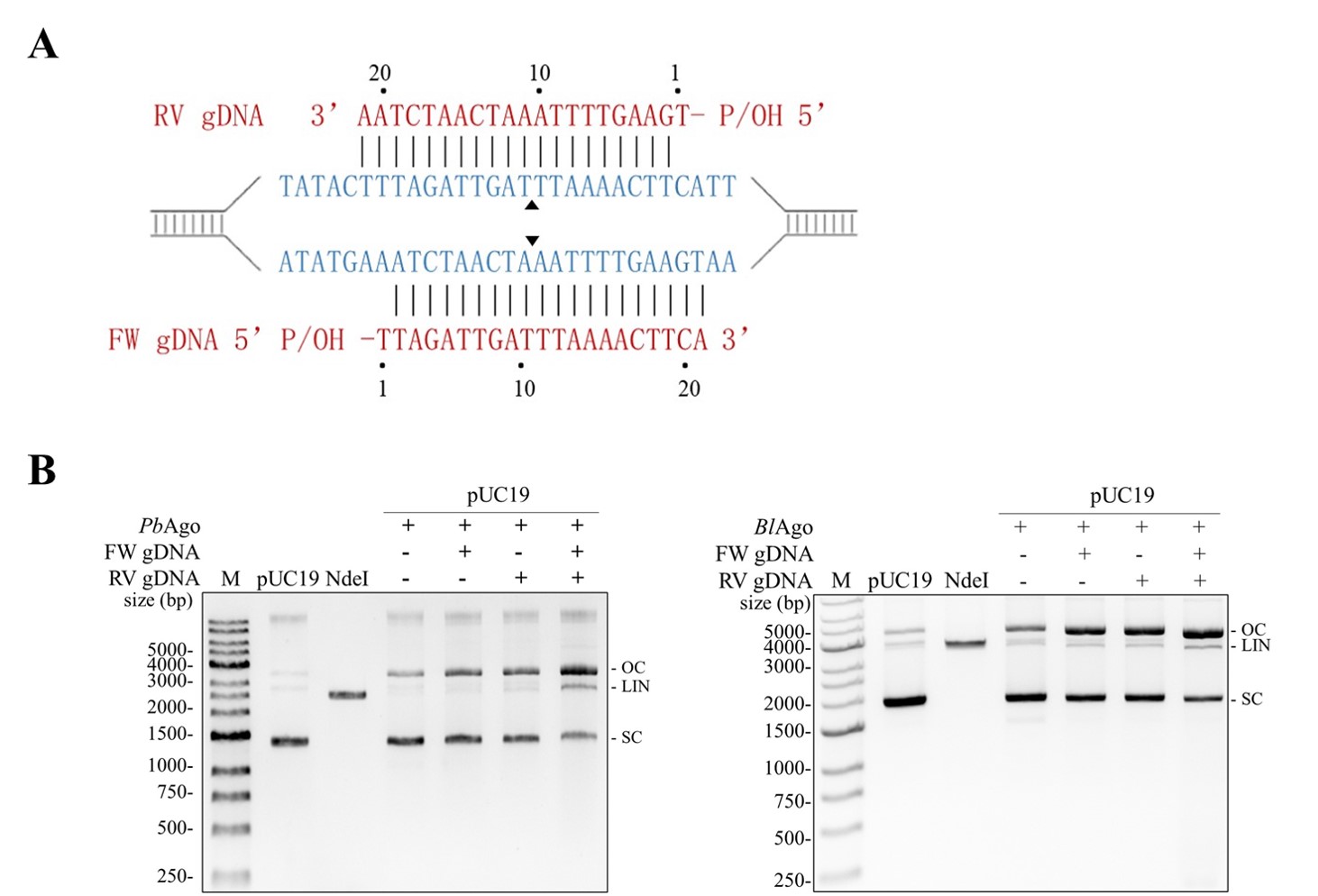


**Fig. S5.** *Pb*Ago and *Bl*Ago cleaves pUC19 guided by 5’OH gDNA. a) Schematic diagram of 5’P/OH DNA guides (red) and pUC19 fragment (blue). Predicted cleavage positions are indicated with a black triangle. b) *Pb*Ago and *Bl*Ago cleaves pUC19 at 65°C guided by 5’OH gDNA.


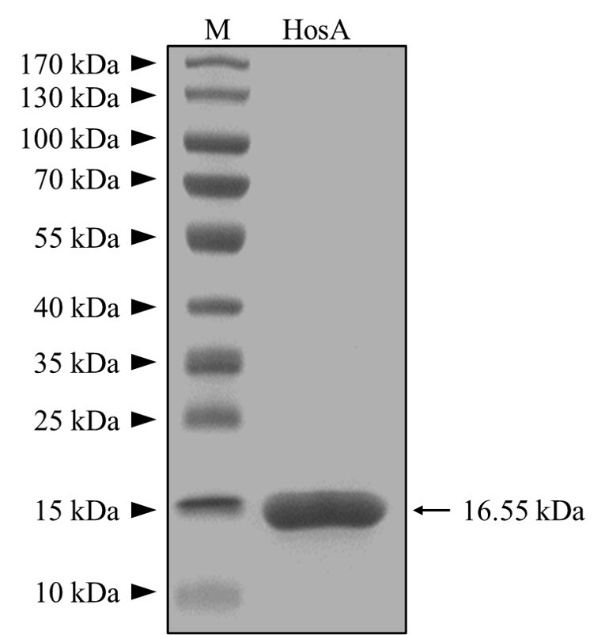


**Fig. S6.** SDS-PAGE analysis of Ni-NTA-purified HosA.

**Supplementary tables**

**Table 1** **Nucleic acids used in ssDNA cleavage**

| **Name** | **Sequence (5’-3’)** | **description** |
| --- | --- | --- |
| ssDNA target | ATATACTATACAACCTACTACCTCGTATAAATTTTTAAATAAATA-FAM | 3’FAM labeled 45 nt ssDNA target |
| ssRNA target | AUAUACUAUACAACCUACUACCUCGUAUAAAUUUUUAAAUAAAUA-FAM | 3’FAM labeled 45 nt ssRNA target |
| 5’P gDNA | P-TGAGGTAGTAGGTTGTATAGT | 21 nt 5’P ssDNA guide |
| 5’OH gDNA | HO-TGAGGTAGTAGGTTGTATAGT | 21 nt 5’OH ssDNA guide |
| 5’P gRNA | P-UGAGGUAGUAGGUUGUAUAGU | 21 nt 5’P ssRNA guide |
| 5’OH gRNA | HO-UGAGGUAGUAGGUUGUAUAGU | 21 nt 5’OH ssRNA guide |
| 78 nt ssDNA target | TCGTAAATAATTTAATATACTATACAACCTACTACCTCGTATAAATTTTTAAATAAATATTGCATTCAAGCTTTTAAT-FAM | 3’FAM labeled 78 nt ssDNA target pair with gDNAs of different lengths |
| 35 nt gDNA | P-TGAGGTAGTAGGTTGTATAGTATATTAAA  TTATTT | 35 nt 5’P ssDNA guide pair with 78 nt ssDNA target |
| 30 nt gDNA | P-TGAGGTAGTAGGTTGTATAGTATATTAAAT | 30 nt 5’P ssDNA guide pair with 78 nt ssDNA target |
| 25 nt gDNA | P-TGAGGTAGTA GGTTGTATAGTATAT | 25 nt 5’P ssDNA guide pair with 78 nt ssDNA target |
| 18 nt gDNA | P-TGAGGTAGTAGGTTGTAT | 18 nt 5’P ssDNA guide pair with 78 nt ssDNA target |
| 16 nt gDNA | P-TGAGGTAGTAGGTTGT | 16 nt 5’P ssDNA guide pair with 78 nt ssDNA target |
| 15 nt gDNA | P-TGAGGTAGTAGGTTG | 15 nt 5’P ssDNA guide pair with 78 nt ssDNA target |
| 14 nt gDNA | P-TGAGGTAGTAGGTT | 14 nt 5’P ssDNA guide pair with 78 nt ssDNA target |
| 13 nt gDNA | P-TGAGGTAGTAGGT | 13 nt 5’P ssDNA guide pair with 78 nt ssDNA target |
| 12 nt gDNA | P-TGAGGTAGTAGG | 12 nt 5’P ssDNA guide pair with 78 nt ssDNA target |
| 10 nt gDNA | P-TGAGGTAGTA | 10 nt 5’P ssDNA guide pair with 78 nt ssDNA target |
| 8 nt gDNA | P-TGAGGTAG | 8 nt 5’P ssDNA guide pair with 78 nt ssDNA target |
| T-gDNA | P/OH -TGAGGTAGTAGGTTGT | 16 nt 5’P/OH ssDNA guide containing 5’T |
| 16 nt 5’P/OH-A ssDNA guide | P/OH -AGAGGTAGTAGGTTGT | 16 nt 5’P/OH ssDNA guide containing 5’A |
| 16 nt 5’P/OH-C ssDNA guide | P/OH -CGAGGTAGTAGGTTGT | 16 nt 5’P/OH ssDNA guide containing 5’C |
| 16 nt 5’P/OH-G ssDNA guide | P/OH -GGAGGTAGTAGGTTGT | 16 nt 5’P/OH ssDNA guide containing 5’G |
| 16 nt 5’P ssDNA guide m1 | P-AGAGGTAGTAGGTTGT | guide forms mismatched pair in position 1 with 45 nt ssDNA target |
| 16 nt 5’P ssDNA guide m2 | P-TCAGGTAGTAGGTTGT | guide forms mismatched pair in position 2 with 45 nt ssDNA target |
| 16 nt 5’P ssDNA guide m3 | P-TGTGGTAGTAGGTTGT | guide forms mismatched pair in position 3 with 45 nt ssDNA target |
| 16 nt 5’P ssDNA guide m4 | P-TGACGTAGTAGGTTGT | guide forms mismatched pair in position 4 with 45 nt ssDNA target |
| 16 nt 5’P ssDNA guide m5 | P-TGAGCTAGTAGGTTGT | guide forms mismatched pair in position 5 with 45 nt ssDNA target |
| 16 nt 5’P ssDNA guide m6 | P-TGAGGAAGTAGGTTGT | guide forms mismatched pair in position 6 with 45 nt ssDNA target |
| 16 nt 5’P ssDNA guide m7 | P-TGAGGTTGTAGGTTGT | guide forms mismatched pair in position 7 with 45 nt ssDNA target |
| 16 nt 5’P ssDNA guide m8 | P-TGAGGTACTAGGTTGT | guide forms mismatched pair in position 8 with 45 nt ssDNA target |
| 16 nt 5’P ssDNA guide m9 | P-TGAGGTAGAAGGTTGT | guide forms mismatched pair in position 9 with 45 nt ssDNA target |
| 16 nt 5’P ssDNA guide m10 | P-TGAGGTAGTTGGTTGT | guide forms mismatched pair in position 10 with 45 nt ssDNA target |
| 16 nt 5’P ssDNA guide m11 | P-TGAGGTAGTACGTTGT | guide forms mismatched pair in position 11 with 45 nt ssDNA target |
| 16 nt 5’P ssDNA guide m12 | P-TGAGGTAGTAGCTTGT | guide forms mismatched pair in position 12 with 45 nt ssDNA target |
| 16 nt 5’P ssDNA guide m13 | P-TGAGGTAGTAGGATGT | guide forms mismatched pair in position 13 with 45 nt ssDNA target |
| 16 nt 5’P ssDNA guide m14 | P-TGAGGTAGTAGGTAGT | guide forms mismatched pair in position 14 with 45 nt ssDNA target |
| 16 nt 5’P ssDNA guide m15 | P-TGAGGTAGTAGGTTCT | guide forms mismatched pair in position 15 with 45 nt ssDNA target |
| 16 nt 5’P ssDNA guide m16 | P-TGAGGTAGTAGGTTGA | guide forms mismatched pair in position 16 with 45 nt ssDNA target |
| 16 nt 5’P ssDNA guide m1m2 | P-ACAGGTAGTAGGTTGT | guide forms mismatched pair in position 1 and 2 with 45 nt ssDNA target |
| 16 nt 5’P ssDNA guide m2m3 | P-TCTGGTAGTAGGTTGT | guide forms mismatched pair in position 2 and 3 with 45 nt ssDNA target |
| 16 nt 5’P ssDNA guide m3m4 | P-TGTCGTAGTAGGTTGT | guide forms mismatched pair in position 3 and 4 with 45 nt ssDNA target |
| 16 nt 5’P ssDNA guide m4m5 | P-TGACCTAGTAGGTTGT | guide forms mismatched pair in position 4 and 5 with 45 nt ssDNA target |
| 16 nt 5’P ssDNA guide m5m6 | P-TGAGCAAGTAGGTTGT | guide forms mismatched pair in position 5 and 6 with 45 nt ssDNA target |
| 16 nt 5’P ssDNA guide m6m7 | P-TGAGGATGTAGGTTGT | guide forms mismatched pair in position 6 and 7 with 45 nt ssDNA target |
| 16 nt 5’P ssDNA guide m7m8 | P-TGAGGTTCTAGGTTGT | guide forms mismatched pair in position 7 and 8 with 45 nt ssDNA target |
| 16 nt 5’P ssDNA guide m8m9 | P-TGAGGTACAAGGTTGT | guide forms mismatched pair in position 8 and 9 with 45 nt ssDNA target |
| 16 nt 5’P ssDNA guide m9m10 | P-TGAGGTAGATGGTTGT | guide forms mismatched pair in position 9 and 10 with 45 nt ssDNA target |
| 16 nt 5’P ssDNA guide m10m11 | P-TGAGGTAGTTCGTTGT | guide forms mismatched pair in position 10 and 11 with 45 nt ssDNA target |
| 16 nt 5’P ssDNA guide m11m12 | P-TGAGGTAGTACCTTGT | guide forms mismatched pair in position 11 and 12 with 45 nt ssDNA target |
| 16 nt 5’P ssDNA guide m12m13 | P-TGAGGTAGTAGCATGT | guide forms mismatched pair in position 12 and 13 with 45 nt ssDNA target |
| 16 nt 5’P ssDNA guide m13m14 | P-TGAGGTAGTAGGAAGT | guide forms mismatched pair in position 13 and 14 with 45 nt ssDNA target |
| 16 nt 5’P ssDNA guide m14m15 | P-TGAGGTAGTAGGTACT | guide forms mismatched pair in position 14 and 15 with 45 nt ssDNA target |
| 16 nt 5’P ssDNA guide m15m16 | P-TGAGGTAGTAGGTTCA | guide forms mismatched pair in position 15 and 16 with 45 nt ssDNA target |

**Table 2** **Nucleic acids used in dsDNA cleavage**

| **Name** | **Sequence (5’-3’)** | **description** |
| --- | --- | --- |
| 21 nt FW 5’P/OH ssDNA guide | P/OH-TTAGATTGATTTAAAACTTCA | FW ssDNA guide pair with pUC19 |
| 21 nt RV 5’P/OH ssDNA guide | P/OH-TGAAGTTTTAAATCAATCTAA | RV ssDNA guide pair with pUC19 |
| 21 nt FW 5’P ssDNA guide | P-TTAGATTGATTTAAAACTTCA | 14 % GC of target region |
| 21 nt RV 5’P ssDNA guide | P-TGAAGTTTTAAATCAATCTAA | 14 % GC of target region |
| 21 nt FW 5’P ssDNA guide | P-TTAAAACTTCATTTTTAATTT | 20 % GC of target region |
| 21 nt RV 5’P ssDNA guide | P-AAATTAAAAATGAAGTTTTAA | 20 % GC of target region |
| 21 nt FW 5’P ssDNA guide | P-TCTCATGACCAAAATCCCTTA | 36 % GC of target region |
| 21 nt RV 5’P ssDNA guide | P-TAAGGGATTTTGGTCATGAGA | 36 % GC of target region |
| 21 nt RV 5’P ssDNA guide | P-CGTGAGTTTTCGTTCCACTGA | 50 % GC of target region |
| 21 nt RV 5’P ssDNA guide | P-TCAGTGGAACGAAAACTCACG | 50 % GC of target region |
| 21 nt RV 5’P ssDNA guide | P-CCCGGCATCCGCTTACAGACA | 60 % GC of target region |
| 21 nt RV 5’P ssDNA guide | P-TGTCTGTAAGCGGATGCCGGG | 60 % GC of target region |
| 21 nt RV 5’P ssDNA guide | P-CGCCCTGACGGGCTTGTCTGC | 70 % GC of target region |
| 21 nt RV 5’P ssDNA guide | P-GCAGACAAGCCCGTCAGGGCG | 70 % GC of target region |

**Table 3** **Nucleic acids used in detection of p-HBA**

| **Name** | **Sequence (5’-3’)** | **description** |
| --- | --- | --- |
| 60 nt ssDNA(HosA)-F | TACTGAGCCATGTATCCAGGTCATTGCGTTCGTATACGAACAGTAGCTCTGACAGTTCCA | FW ssDNA contain HosA recognition sequence |
| 26 nt ssDNA(HosA)-R | GTCAGAGCTACTGTTCGTATACGAAC | RV ssDNA contain HosA recognition sequence |
| 16 nt 5’P ssDNA guide | P-CGCAATGACCTGGATA | guide pair with 60 nt ssDNA(HosA)-F |
